# Configuration of Mobile Learning Tools to Support Basic Physical Assessment in Nursing Education: Longitudinal Participatory Design Approach

**DOI:** 10.2196/22633

**Published:** 2021-01-07

**Authors:** H Ösp Egilsdottir, Lena Günterberg Heyn, Espen Andreas Brembo, Kirsten Røland Byermoen, Anne Moen, Hilde Eide

**Affiliations:** 1 Science Centre Health and Technology Faculty of Health and Social Sciences University of South-Eastern Norway Drammen Norway; 2 Institute for Health and Society Faculty of Medicine University of Oslo Oslo Norway

**Keywords:** learning, mobile phone, mobile application, education, nursing, students, nursing, education, clinical, nursing skills, physical examination, computer simulation, clinical competence

## Abstract

**Background:**

As many students in higher education are skilled users of mobile technology, mobile learning (mLearning) can be a promising educational strategy to enhance their learning experience. mLearning might also be well suited for nursing students as they navigate between multiple learning contexts in their educational curriculum. As an educational strategy, mLearning may also reduce challenges caused by the theory-practice gap in nursing by supporting skills and knowledge transfer between the university and clinical settings. As the introduction of basic physical assessment skills (B-PASs) into Norwegian bachelor’s degree education in nursing occurred quite recently, there is a lack of competence in supervision and teaching in both university and clinical settings. As such, mLearning appears to be a good strategy to support student B-PAS learning and knowledge transfer across learning contexts.

**Objective:**

This study aims to explore and elicit the perspectives of students regarding the way in which a selection of digital learning resources supports B-PAS learning and application in clinical rotation, which of the selected digital learning resources are beneficial to include in a suite of mLearning tools, and how the selected digital learning resources could support the transfer of skills and knowledge from the academic to clinical context.

**Methods:**

We used a longitudinal participatory design approach to co-design a suite of mLearning tools. The co-design processes took place in several workshops (WSs) over a period of 3 months: 2 WSs with first-year students (n=6), 3 WSs with second-year students (n=6), and 3 WSs with third-year students (n=8). The students evaluated several digital learning resources in both academic and clinical contexts. The digital learning resources included digital simulation with virtual patients, massive open online courses, and multimedia learning material. In the co-design WS, the potential and benefits of these digital learning resources for the learning and application of B-PASs were explored.

**Results:**

The students reported that the digital learning resources stimulated learning in 7 different ways. They also emphasized the importance of including all selected and tested digital learning resources. Moreover, students supported the inclusion of additional learning material, such as multiple-choice tests and written assignments, aimed at providing feedback and contributing to knowledge development.

**Conclusions:**

The co-design processes and collaboration with the nursing students provided insight into how a suite of mLearning tools may support the learning and application of B-PASs and human bioscience knowledge in clinical rotation. From the students’ perspective, one of the strengths of the suite of mLearning tools was the range of content, as this met a broader range of student learning preferences regarding learning B-PASs. The suite of mLearning tools contributes to and supports skills training and knowledge transfer between multiple learning contexts.

## Introduction

### Mobile Learning Opportunities in Higher Education

Students pursuing higher education today expect teaching methods that stimulate self-directed learning, active learning, peer learning, and the cocreation of knowledge [1,2]. Many of these students belong to the *digital natives* generation having grown up with mobile technology such as smartphones; this makes them skilled users, especially with regard to social interaction and finding information on the web [2,3]. Mobile technology used for mediating educational content offers flexibility in teaching methods; thus, mobile learning (mLearning) promotes and contributes to learning processes that are less constrained by time and context [4-6]. Research indicates that mLearning contributes to meaningful and comprehensive learning experiences, inviting students to select their preferred modalities and share the responsibility for their own learning processes [1,7,8].

In nursing education, students navigate between multiple learning contexts during lectures and different clinical rotation periods. This requires students to handle and mitigate the differences between what is taught in universities and what they learn in the clinical rotation—this is commonly referred to as the theory-practice gap [9]. mLearning is shown to be a good strategy for reducing these challenges by supporting students in becoming less dependent on the learning context and increasingly self-directed in their own learning processes [10,11]. When considering the implementation of a suite of mLearning tools in nursing education, students should be core collaboration partners because of their role as end users [11,12]. Importantly, as nursing students are the ones who must navigate between multiple learning contexts, they should be the ones evaluating and testing mLearning and its possible content to determine what works for them and in what context [11]. As such, a participatory design involving co-design workshops (WSs) is a good method to highlight end user involvement and collaboration in the development and evaluation of the end product [12].

### Rationale for Educational Focus in a Suite of mLearning Tools

Nurses base their clinical judgment on physical assessment and history taking to map their patients’ health condition [13]. Physical assessment skills (PASs) are therefore one of the core competencies nurses must master [14]. Inadequate patient assessment might result in a failure to notice deteriorating patients and to initiate appropriate nursing interventions; this can threaten the safety of patients and result in adverse health outcomes [15,16]. However, performing adequate patient assessments can be challenging for novice nurses because of the complexity of health situations in the different patient groups that they encounter [16]. The nursing students learn PAS in preclinical courses at the university but apply these skills in clinical rotation. Therefore, learning and developing competence and confidence in performing PAS and the ability to articulate relevant knowledge are based on how these skills are taught on campus and on the supervision of preceptors during the clinical rotation [16,17].

**Figure 1:**
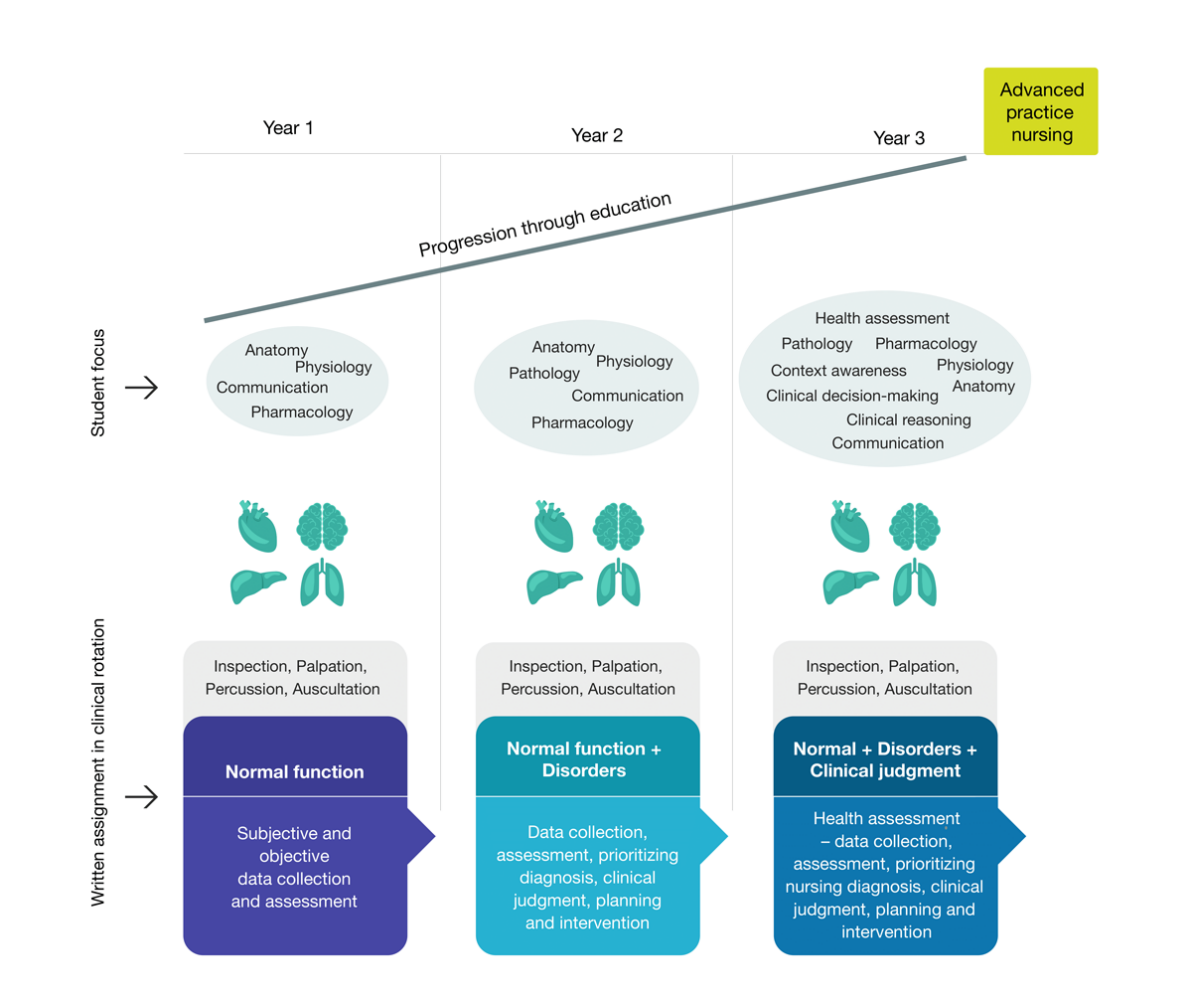
Basic physical assessment skills progression model in Norwegian nursing education.

Until recently, PASs were not a part of nursing education in Norway. To overcome the barriers identified in international studies [14], our university implemented selected PASs focusing on respiratory, peripheral circulation, abdominal, and neurological assessments in the curriculum in 2015. These PASs are considered to be the basic skills necessary for clinical competence for undergraduate nursing students and are, therefore, referred to as *Basic* PAS (B-PAS; Figure 1) [17].

In a previous study, we identified that a lack of knowledge and practical use of B-PASs among the faculty and preceptors limited students in their learning of and performing these skills [17]. This points to a theory-practice gap with regard to developing these specific skills and underlying knowledge. This is concerning, as 50% of Norwegian nursing education takes place in clinical rotations in different contexts [18]. An additional result from this earlier study was that students identified mLearning as potentially enhancing their B-PAS learning processes. This finding is supported by other studies [19,20]. Research also highlights that mLearning can contribute to the articulation and integration of human bioscience knowledge (anatomy, physiology, pathophysiology, and pharmacology), which underpins knowledge-based basic physical assessment.

Thus, mLearning can support nursing students in learning and applying B-PASs in a clinical context without being solely dependent on guidance from their supervisors. mLearning may also contribute to better transfer of knowledge between multiple learning contexts, and it arguably works best in combination with other teaching methods and educational strategies [21-24].

There is a range of digital learning resources that are suitable for inclusion in a suite of mLearning tools highlighting B-PASs and relevant knowledge, for example, massive open online courses (MOOCs) [25], web-based apps, gaming [22], podcasts [26], multimedia presentations [27], and more comprehensive software involving immersive technology, such as digital simulation with virtual patients [28,29]. Digital simulation involves real people using digital and mobile technology to operate interactive virtual patient scenarios for educational purposes [30]. When reviewing the literature regarding factors facilitating or hindering the implementation of mLearning in medical and nursing education, Lall et al [31] not only identified high student satisfaction with mLearning but also revealed challenges related to the implementation process. These challenges existed both in universities and clinical settings and were related to faculty competence in using mobile technology, the acceptance of using mobile devices in different clinical settings, and students’ access to the devices [31].

Therefore, the aim of this study is to co-design a suite of mLearning tools with nursing students to support their B-PAS learning and application. The specific objectives are to explore the following with students: (1) in what way does the selection of digital learning resources support B-PAS learning and application in clinical rotation; (2) which of the selected digital learning resources are beneficial when included in the suite of mLearning tools; and (3) how the selected digital learning resources could support the transfer of knowledge from academic context to clinical settings.

## Methods

### Research Design

This qualitative longitudinal study was inspired by participatory design. Participatory design aims to involve end users in co-design and development processes with a specific purpose [12]. The co-design processes took place in several iterative WSs over a period of 3 months: 2 WSs with first-year students (n=6), 3 WSs with second-year students (n=6), and 3 WSs with third-year students (n=8). Through these WSs, the experiences and preferences of students were explored with the aim of informing the final selection of digital learning resources included in the suite of mLearning tools.

### Recruitment and Sample

The nursing students were recruited from a bachelor’s program in nursing at a large university in Norway. Information about the aim of this study and an invitation to participate were published on the learning management system (LMS) of the university, and a short presentation about this study was given orally on campus. Students signed up to participate by emailing the first author (ÖE) directly or via their course leader. The convenience sample consisted of 20 nursing students (14 women and 6 men) in the first, second, and third year of the program. The participants’ ages varied from 20 to 50 years.

### Procedure and Data Collection

All the WSs were held in 2019 during the spring semester on the university campus. They were facilitated by the first author (ÖE), who was supported by one of the other researchers (EB, LH, or HE) during each WS. ÖE was also an active participant in the discussions. The main tasks for the facilitators were to involve all the students in the discussions, ensure that all planned topics were covered, and keep track of time and breaks. Each WS lasted a maximum of 2 hours, and the discussions were audio recorded. WSs 1 and 2 were transcribed verbatim, whereas WS 3 was worked with as audio files in which important statements were marked.

### The Digital Learning Resources Suggested as Content in the Suite of mLearning Tools

**Figure 2:**
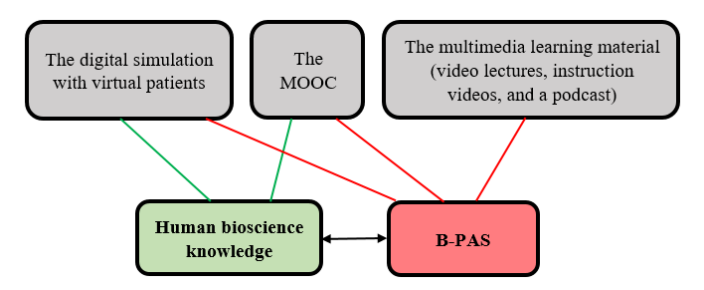
Learning focus for the different digital learning resources tested by the students. B-PAS: basic physical assessment skills; MOOC: massive open online course.

A selection of digital learning resources highlighting B-PASs in different ways were introduced to and assessed by the participating nursing students. The digital learning resources included (1) a digital simulation program with virtual patients, (2) a MOOC, and (3) multimedia learning material (video lectures, instruction videos, and a podcast; Figure 2). These digital learning resources were new to most of the students.

The digital simulation program consists of several interactive virtual patient cases and can be used in groups or as an individual learning activity. One of the tasks in the digital simulation is to choose appropriate B-PASs and other interventions during assessment of virtual patients; the students also received feedback on their choice of assessments and the interventions performed in the program—fitting perfectly with the aim of the suite of mLearning tools. The MOOC is an individual e-learning course with 5 modules promoting students’ assessment of vital signs and history-taking skills, notice of patient deterioration processes, and learning about more advanced skills (eg, auscultation of the lungs and heart). In addition, it was desirable to evaluate the selection of multimedia material, consisting of video lectures, instruction videos, and a podcast, all aiming to teach (verbally and visually) how to perform B-PASs on real patients. The podcast focused on giving the students perspectives on how to learn and focus on using these skills throughout their nursing education.

### Content and Structure of Co-Design WSs

**Figure 3:**
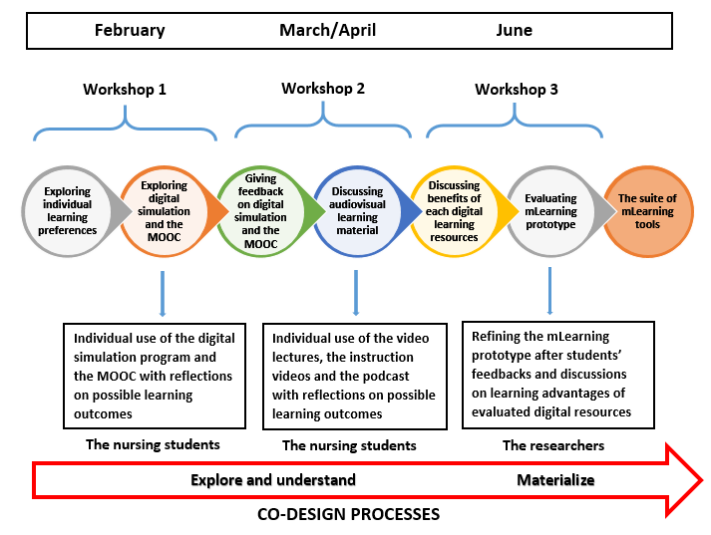
Co-design processes in the workshops. mLearning: mobile learning; MOOC: massive open online course.

The researchers planned and framed the structure and content of the 3 WSs, which were generally based on the selected digital learning resources. Students engaged in the co-design processes by using and evaluating how the digital learning resources influenced their learning related to B-PASs and human bioscience knowledge during clinical rotation and in academic courses. Figure 3 provides an overview of the processes in the co-design WSs.

The first WS included the introduction and discussion of 2 new learning resources: the digital simulation with virtual patients and the MOOC. We asked the students to evaluate and test these 2 digital learning resources individually before attending the second WS. In the second WS, we explored the experiences of students with using digital simulation and the MOOC. As B-PASs is built on knowledge of human biosciences, it was important to discuss with the students as to what type of learning material or assignments could facilitate coherence between theoretical knowledge of human biosciences and the application of these skills. The nursing students were asked to use and evaluate the multimedia learning materials (videos and podcast) before attending the next WS. In the third WS, we focused on discussing the students’ feedback regarding the multimedia learning materials. Here, it was important to discuss the digital learning resources to be included and the ones to be excluded. In addition, we presented a pilot version of the suite of tools to give the students a visual example of how they might look in the LMS. This way, the students had the opportunity to give feedback and input regarding the first prototype. The participation of nursing students was relatively stable throughout the WSs; however, some students participated in only 1 WS (Table 1).

**Table 1:** Participation of nursing students in the workshops.

Student			WS^a^ 1		WS 2		WS 3
**First-year students**							
	Student 1	✓^b^		—^c^		—	
	Student 2	✓		—		—	
	Student 3	✓		✓		—	
	Student 4	✓		—		—	
	Student 5	✓		—		—	
	Student 6	—		✓		—	
**Second-year students**							
	Student 7	✓		✓		✓	
	Student 8	✓		✓		✓	
	Student 9	✓		✓		✓	
	Student 10	✓		—		—	
	Student 11	✓		✓		✓	
	Student 12	—		✓		✓	
**Third-year students**							
	Student 13	✓		✓		—	
	Student 14	✓		✓		✓	
	Student 15	✓		✓		✓	
	Student 16	✓		✓		✓	
	Student 17	✓		✓		✓	
	Student 18	✓		✓		—	
	Student 19	✓		✓		✓	
	Student 20	—		✓		—	

^a^WS: workshop.

^b^Attending the workshop.

^c^Not attending the workshop.

### Research Ethics

The Norwegian Centre for Research Data approved the study (Reference number 462735). Written informed consent was obtained from all participants in accordance with the Declaration of Helsinki. Reviewing the learning resources was not a requirement to attend WSs 2 and 3. The researchers were not involved in evaluating the academic or clinical courses of the participants, and participation in this study had no impact on grading in any of the courses.

### Data Analyses

**Figure 4:**
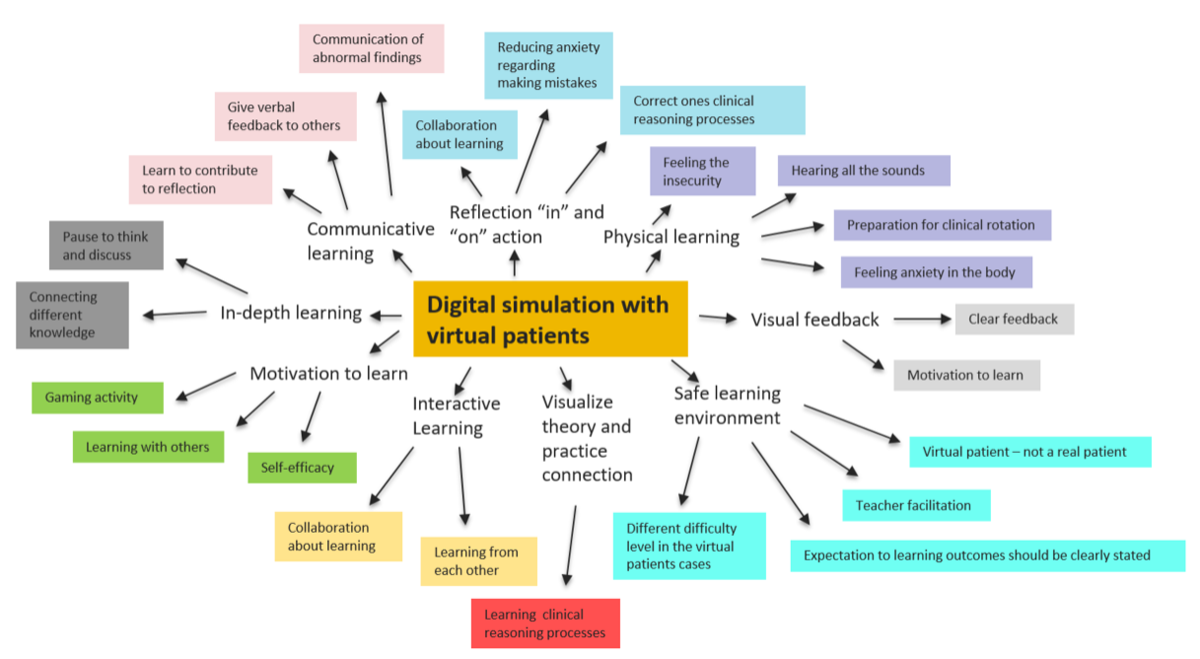
Example of a concept map in the analysis process.

Thematic content analysis was used to analyze data [32], which comprised text materials (WSs 1 and 2) and audio files (WS 3). The research questions guided this process. The text material from WSs 1 and 2 (all student cohorts) and the audio files from WS 3 (second- and third-year students) were read and listened to completely, respectively. Further analysis of the data included important concepts and statements involving the design of and suggested content in the suite of mLearning tools. Figure 4 shows an example of how the digital resources (in this case, the digital simulation with virtual patients) were creatively mapped to explore core concepts throughout the analyses of the data.

## Results

**Figure 5:**
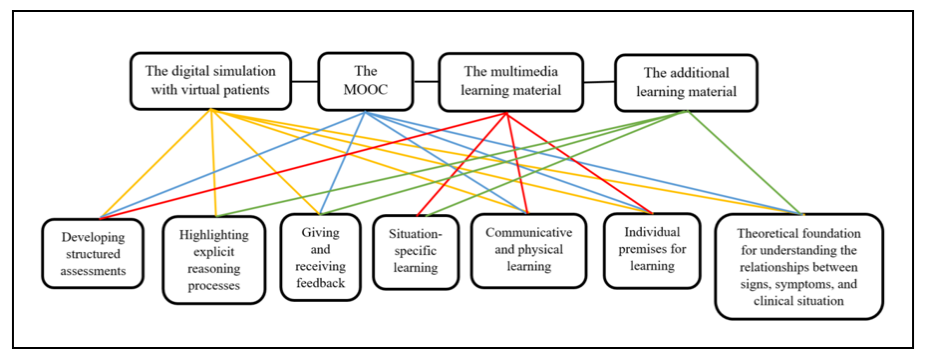
Contribution of digital learning resources to student learning. MOOC: massive open online course.

According to the students, the digital learning resources stimulated learning in 7 different ways: (1) developing structured assessment, (2) highlighting explicit reasoning processes, (3) giving and receiving feedback, (4) situation-specific learning, (5) communicative and physical learning, (6) individual premises for learning, and (7) the theoretical foundation for understanding the relationships between signs, symptoms, and clinical situation. Figure 5 shows how the digital learning resources contributed to student learning, both individually and collectively.

In the following sections, each digital learning resource and its influence on the learning process are presented. Students’ own voices, in the form of quotations, are included in Textbox 1 to substantiate the data analyses. The content of the suite of mLearning tools is then presented.

**Students’ quotations related to the evaluation and testing of digital learning resources; these quotes substantiate concept mapping and data analysis.**

- The digital simulation with virtual patients:
  Student 13 workshop (WS) 1: “I believe that this (digital simulation) will be very attractive for the students, it’s a really cool way to learn and the students might be more motivated to learn anatomy and physiology, rather than sitting for six to seven hours listening to a lecture, which is really exhausting”
  Student 17 WS 2: “There is always something that goes wrong. Then you have the opportunity to pause the simulation and review what you missed or what you did wrong. Luckily it is a fictive patient but you learn in what order you should do things”
  Student 8 WS 1: “We get the opportunity to meet someone really ill in a totally safe environment”
- The Massive Open Online Course
  Student 15 WS 3: “If the solution of the case is a stroke diagnosis and you didn’t think of that, then there are obviously some really important cues that you missed”
- Multimedia learning materials
  Student 14 WS 3: “It is really important to show the reasoning processes—it’s not important what the conclusion is but just to show the reasoning processes”
  Student 11 WS 2: “It would be perfect if lectures were available online. If you don’t understand certain things and you are afraid to ask in the classroom, then you can just rewind and look at the material that is difficult to understand. You can also have the textbook beside you to try to figure it out”
  Student 2 WS 2: “In order to recognize potential symptoms of a disease, you must know the normal body functions. Then you must know what to listen for—you cannot just listen to the lungs without knowing. It requires fundamental theoretical knowledge”
- Additional learning materials
  Student 7 WS 3: “I would rather use my time and effort on something that gives me feedback on or an indication of my knowledge base, than on academic assignments in clinical rotation where I never get any feedback at all”
  Student 15 WS 3: “There is something about being conscious regarding one’s own feedback to other people. To be conscious about what word you use and how to give constructive feedback—and we will have to do that when we become supervisors for nursing students later”
- General design features for better end user experience
  Student 11 WS 1: “A multimedia learning resource is an extremely effective learning tool within some courses—it is all about experimenting and finding out what works. But a well-designed digital learning resource can help to show how different knowledge is intertwined”

### Digital Simulation With Virtual Patients

In general, all the students were excited about the digital simulation and the possibilities that this learning resource could offer for skills and knowledge development. The first-year students discussed how digital simulation could enhance their learning of human bioscience knowledge and the ability to connect it with other topics in nursing education. The second- and third-year students highlighted its usefulness related to the development of communication and clinical reasoning skills, as they learned to prioritize nursing interventions and focus on performing B-PASs. The nursing students also elaborated on their physical reactions in the situation. The sounds from the patient (eg, moaning in pain) and instruments (eg, changes in vital signs) made the students feel stressed, as they would have in a real patient situation. With this authenticity, the students felt that the digital simulation with virtual patients would contribute to physical and mentally preparedness for clinical rotation. The use of English in the digital simulation software created some challenges for most of the students; as such, they highlighted the value of working in their native language with regard to improving learning outcomes and the overall user experience.

After testing the digital simulation between WSs 1 and 2, the students emphasized the benefits of working together in a group rather than working with the virtual patient cases individually. The students also described how faculty was important when working in a group in the digital simulation sessions, with regard to promoting a safe learning environment, facilitating knowledge development, and collaborating within the group. The safe learning environment was viewed as a prerequisite for students’ reflections *in* action and *on* action, influencing each other’s learning processes by making it *safe* to actively participate in a discussion with one’s peers.

Another important factor in the digital simulation was the opportunity to learn from making incorrect or suboptimal decisions without harming a real patient during the simulation. To enhance this learning outcome, the process of reflection during the simulation session and in the debriefing phase were crucial to understanding what went wrong. Through the digital simulation, the students also learned the importance of performing structured physical assessments; this included learning where to start gathering data for a thorough overview of the patient situation to initiate nursing interventions.

The nursing students also stressed that the digital simulation with virtual patients used in combination with theoretical classes and during clinical rotation periods might be a beneficial pedagogical strategy to enhance learning, especially in theoretical classes involving human bioscience knowledge. Some of the students in all 3 cohorts drew parallels to gaming experiences and discussed ways these may be helpful in the use of the digital simulation.

### The MOOC

The structure of the 5 modules included in the MOOC appealed to the students. They found that elder care or community health care was the primary focus and suggested that the MOOC was best suited to the curriculum in the first and third years (in the Norwegian context). As some of the assignments were built on one specific clinical case, the students felt that a broader selection of cases would provide better knowledge development. The second- and third-year students especially liked how communication skills were emphasized in the MOOC. In their opinion, focusing on communication skills is as important as focusing on technical skills during clinical rotation. The nature of the assignments, such as listening to audio files, appealed to the second- and third-year students. Some of the assignments also required that they categorize patient data into objective or subjective data. In this manner, the MOOC supported students’ learning regarding the value of working in a structured way when prioritizing data collection in clinical cases. Moreover, the clinical cases supported the cognitive processes involved in *catching the cues*, and for students, this was an important learning outcome of the MOOC.

### Multimedia Learning Materials

The multimedia materials included video lectures, instruction videos, and a podcast. The students highlighted that the use of these learning materials supported different clinical situations, enabling them to selectively choose specific learning material before performing B-PASs with the patient. According to the students, this type of learning material can contribute to both knowledge development and self-efficacy. They found it helpful to have the learning outcomes clearly stated regarding the expected level of B-PAS performance. Students also valued the podcast format, which could be used to discuss clinical cases showing experienced nurses’ clinical reasoning processes in action.

The students valued the functional possibilities offered by the multimedia materials, for example, they could pause the audio files to familiarize themselves with difficult concepts and then replay the audio files as often as necessary. Here, the auscultation skills were highlighted as especially challenging, particularly relating to the interpretation of lung and heart sounds. The students also suggested that the features in the multimedia materials could be simple animations, not necessarily actual patients, as the students have access to real patients during clinical rotation. Although they thought the use of animations instead of actual patients might contribute to a better understanding of the connection between human biosciences and the clinical situation, the students highlighted that the learning materials must be of high (sound and picture) quality.

### Additional Learning Materials

The students found it helpful to include additional learning materials aimed at supporting knowledge transfer in and development of the suite of mLearning tools. This additional content consisted of tests with multiple-choice questions (MCQs), several clinical cases to choose among, and written assignments. The students also emphasized the inclusion of clearly stated learning outcomes related to this additional material, making it easier to understand the value of engaging with the learning material. They felt this could also contribute to knowledge development and self-efficacy. The students wanted access to the correct answers for the MCQ tests and the possible solutions for the clinical cases: they argued that this type of immediate feedback could help them understand their own level of performance and identify knowledge gaps. The students also suggested that it was important, for purposes of motivation and self-efficacy, to receive the results or feedback visually—perhaps with audio animation or a pop-up effect.

The students gave suggestions regarding how to structure this part of the suite of mLearning tools in a way that would trigger their curiosity and motivation to learn more. One suggestion was that correct answers in MCQ tests or clinical cases could *unlock* further advanced learning material. The students welcomed a written assignment targeting reflection on using the B-PAS during clinical rotation, preferably with a peer review; they felt that this would also provide them with experience in providing critical and constructive feedback. They did not want open peer review but preferred anonymity because of the different relationships between students and their own insecurities regarding giving peer feedback.

### General Design Features for Better End User Experience

One important element of the co-design processes concerns the general design features that emerged from the WS discussions. The students emphasized that the suite of mLearning tools had to be easily accessible, have a logic structure, be compatible with smartphones, and be usable *on the go*. Students highlighted the importance and possibilities of linking the suite of mLearning tools to other relevant web-based resources. The second- and third-year students recommended limited access for first-year students to avoid overwhelming them with too much information. One solution that they suggested was to design a *lock* in the structure of the suite of mLearning tools that could be unlocked by the most motivated first-year students who are eager to learn more. It was also important for the students to have the possibility of being anonymous or to use avatars if the use of the tool was visible to other students.

### The Suite of mLearning Tools Recommended From the Co-Design WSs

The co-design processes included all selected digital learning resources in the suite of mLearning tools aiming to support the B-PAS and knowledge development. The LMS of the university was used to structure the suite of mLearning tools, which enabled the students to access digital learning resources by using mobile devices. Table 2 provides an overview of the content available to the students and a short description of the different digital resources. The content in the LMS had no fixed order, enabling students to access the content in which they were specifically interested. The co-design processes and the longitudinal research design allowed students to test and evaluate the selected digital resources when in different learning contexts, for example, in an academic context and in a clinical rotation context. In such cases, the student expressed that this suite of mLearning tools and the future use of mobile technology may have teaching and learning value in both theoretical and practical courses—a valuable educational component during challenging situations such as the current COVID-19 pandemic.

There was a small difference in what was planned for the first-year students to have access to. The written assignment was included for all years, but the element of peer review was excluded for the first-year students based on an assessment made by the older students and the research group. As none of the first-year students were able to attend the final WS, there was no opportunity to discuss with them the potential advantages or disadvantages of peer review. According to the second- and third-year students, the main strength of the suite of mLearning tools was that it offered different types of digital learning resources from which to choose. Thus, these students found it useful to include all of the selected and evaluated digital learning resources in the suite of mLearning tools.

**Table 2:** Overview of the structure and content of the suite of mobile learning tools in the learning management system.

Different digital learning resources	Elaboration of the content
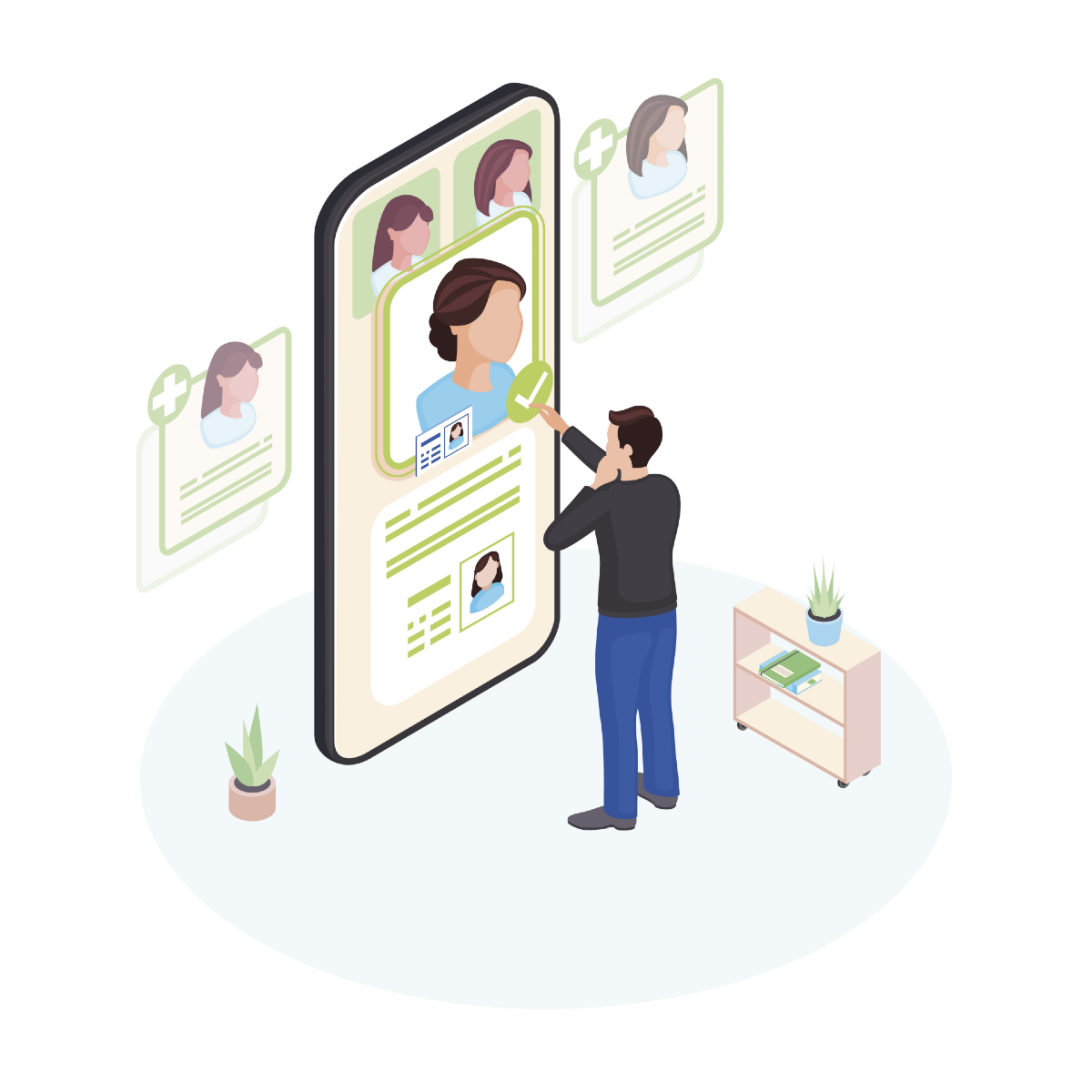	A short description of the digital simulation program, information about the sessions, and the virtual patient cases on which students can work. Also included are log-in details and information about whom to contact if there are problems with the log-in
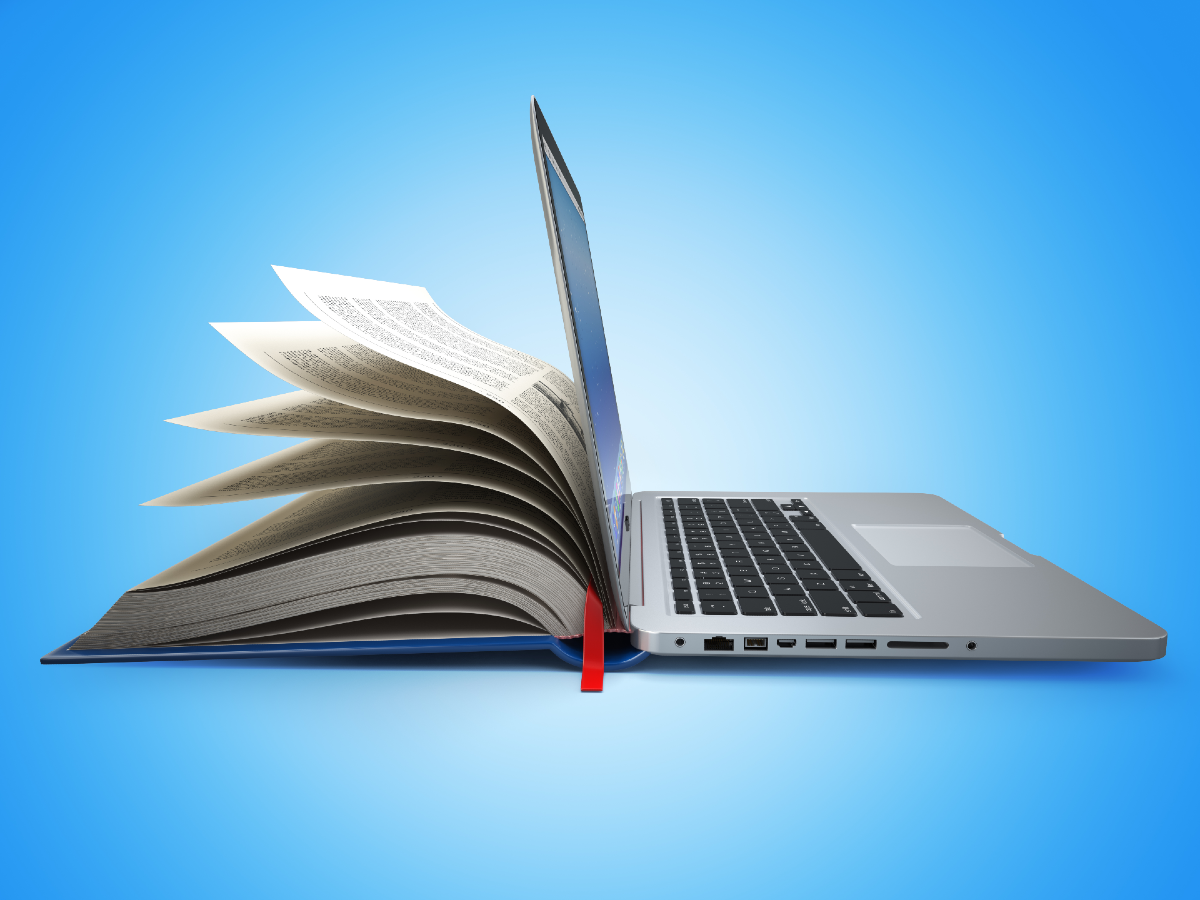	A brief description of the MOOC^a^ and recommendation for which modules students should focus on, depending on which educational year they belong to. The students also find details here regarding the log-in process
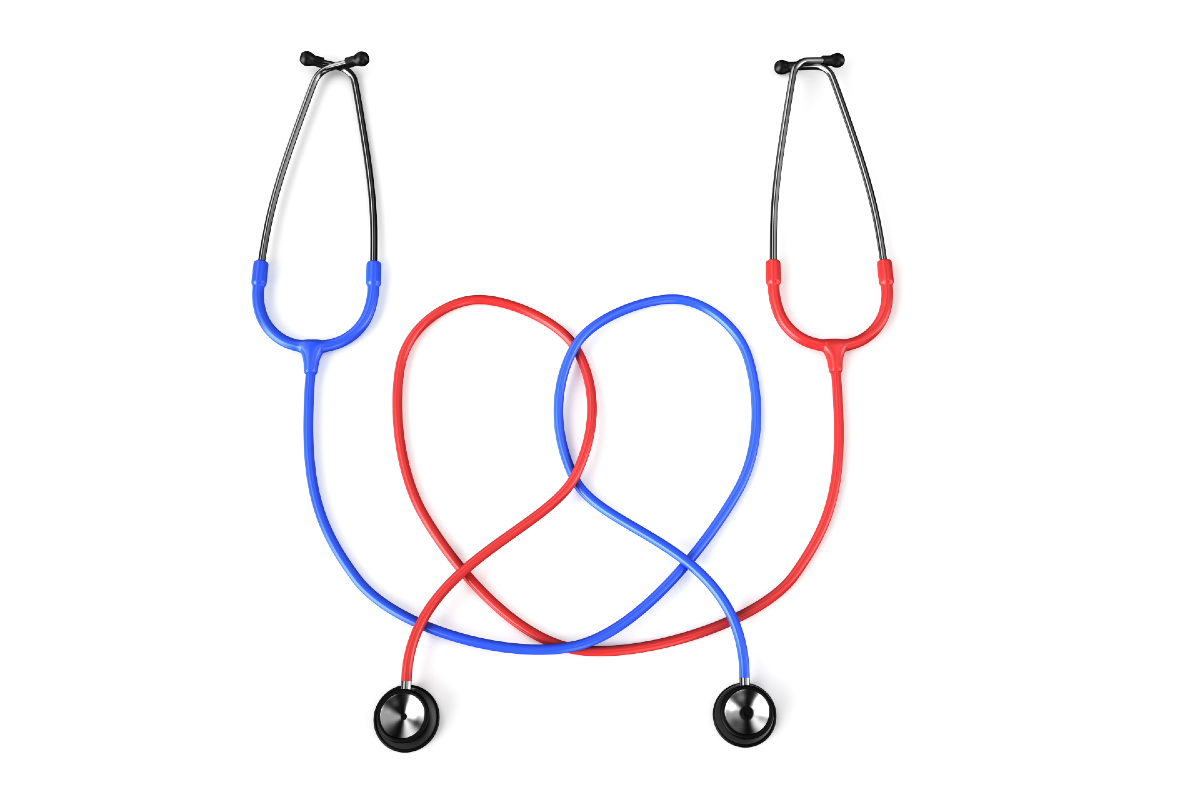	Detailed information about auscultation skills, divided into 2 sections: (1) lung sounds and (2) heart sounds. Each section contains links to YouTube videos and audio files with different sounds to which students may listen
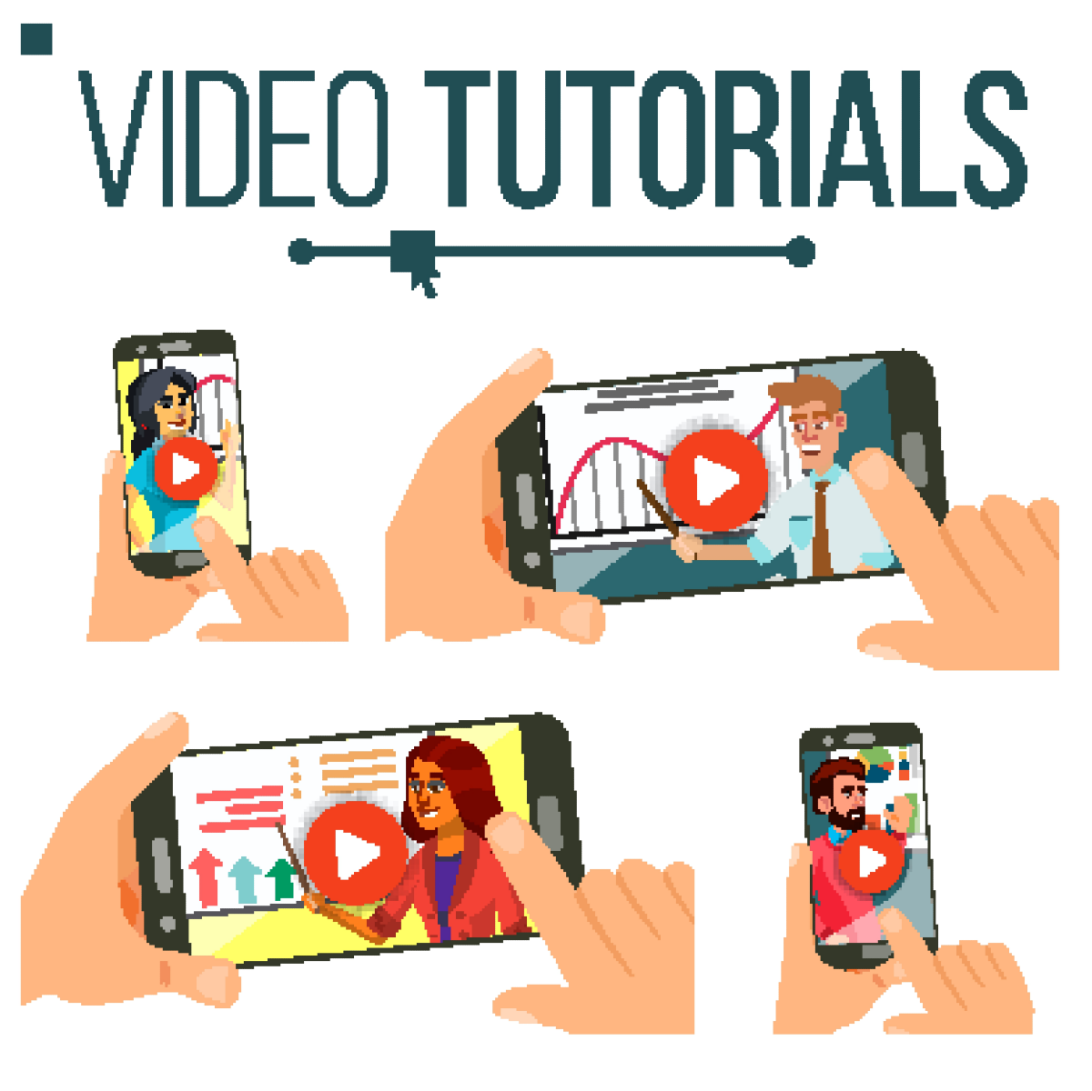	A total of 5 instruction videos with a nurse performing B-PASs^b^ on a patient. Each video has a specific focus: the heart and peripheral circulation, the respiratory system, the abdomen, the neurological assessment, and recording vital signs. The duration of the videos is from 7:37 to 15:51 min
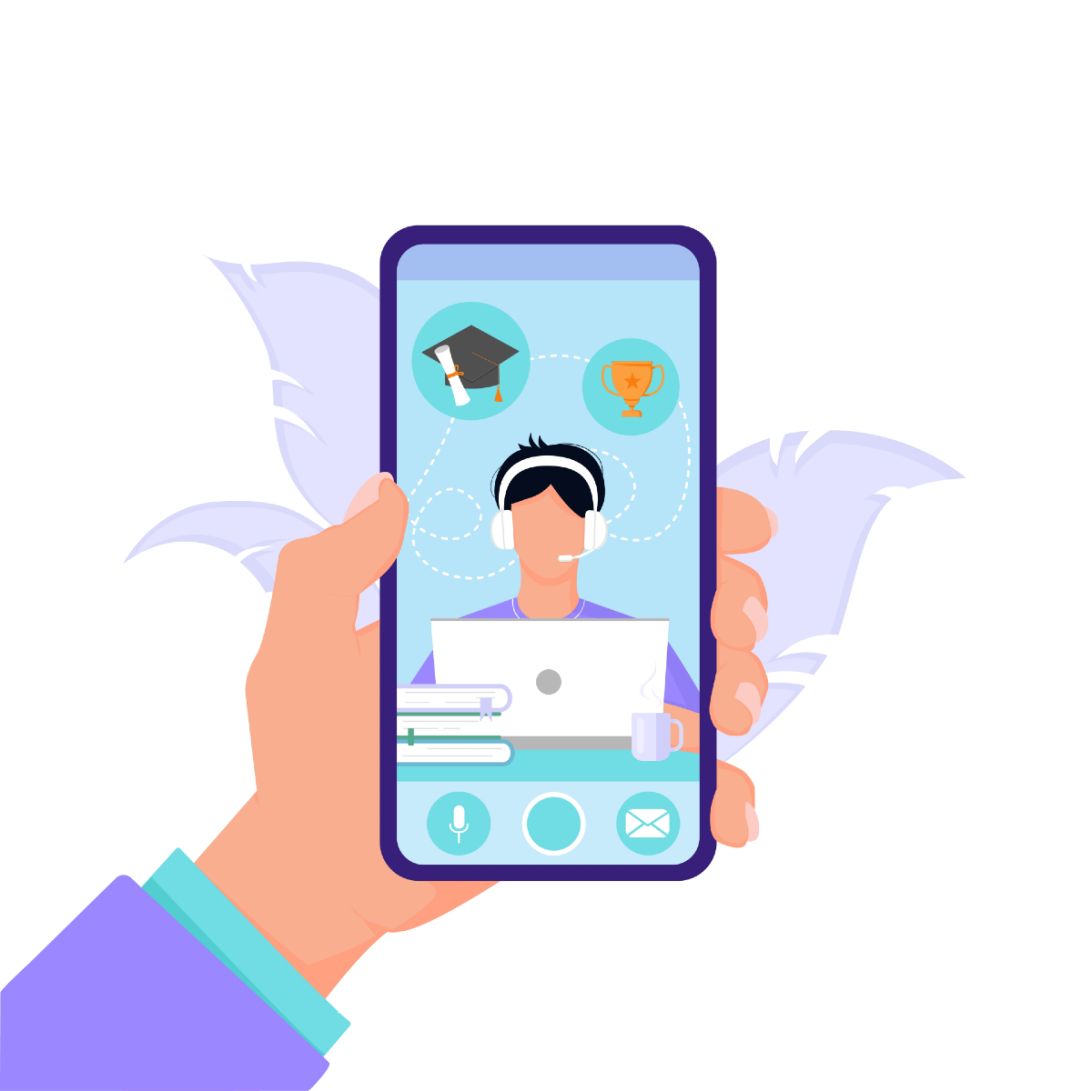	A total of 4 video lectures in which each video has a specific focus: the heart and peripheral circulation, the respiratory system, the abdomen, and the neurological assessment. The duration of the videos is from 13:13 to 32:55 min
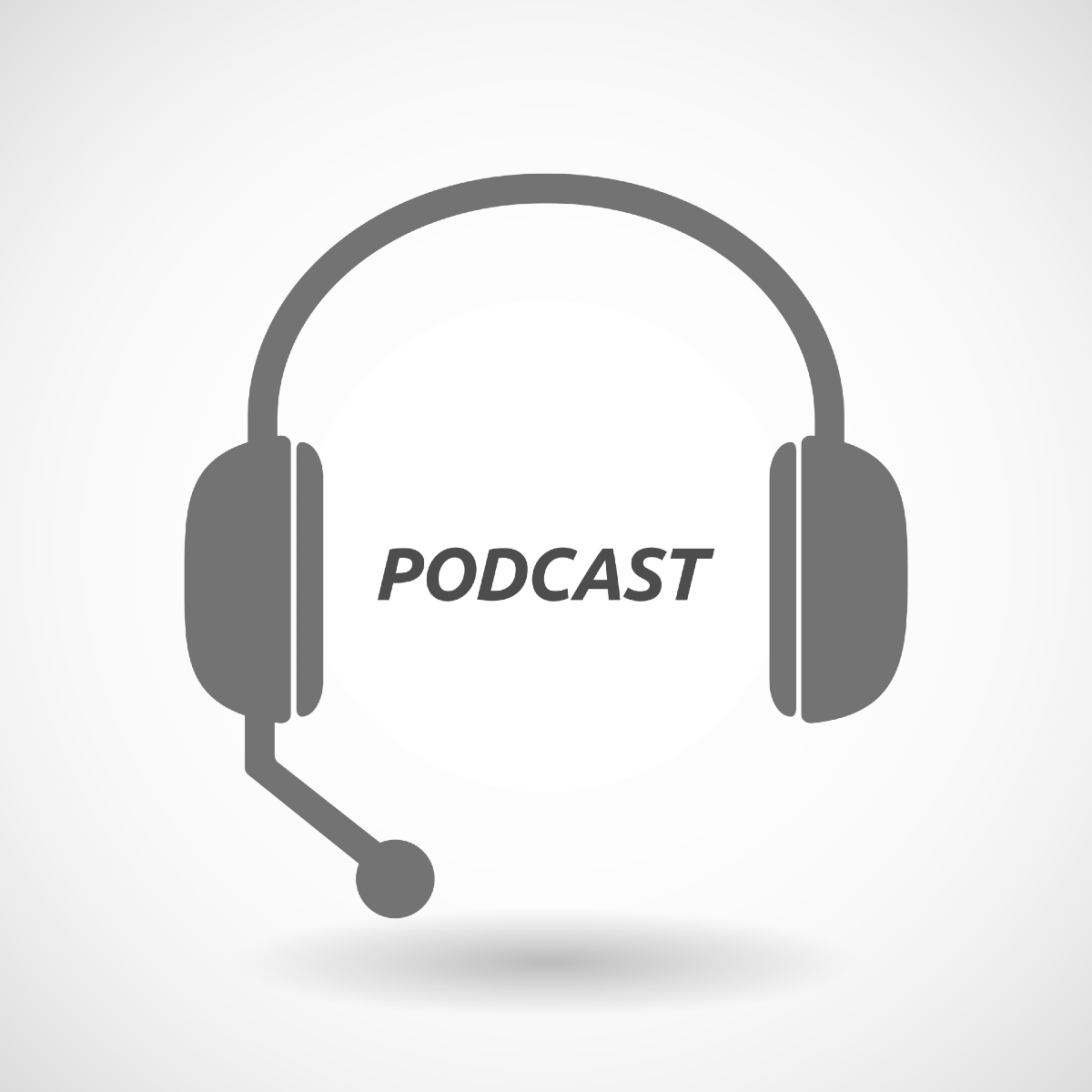	Two nurses (faculty members) talk about the origin of the physical assessment in nursing education and the differences between performing B-PASs as a RN^c^ or as a nurse practitioner A conversation between the faculty members and 2 newly graduated RNs, focused on working with B-PASs throughout the 3-year nursing program, and how they work with B-PASs as new RNs
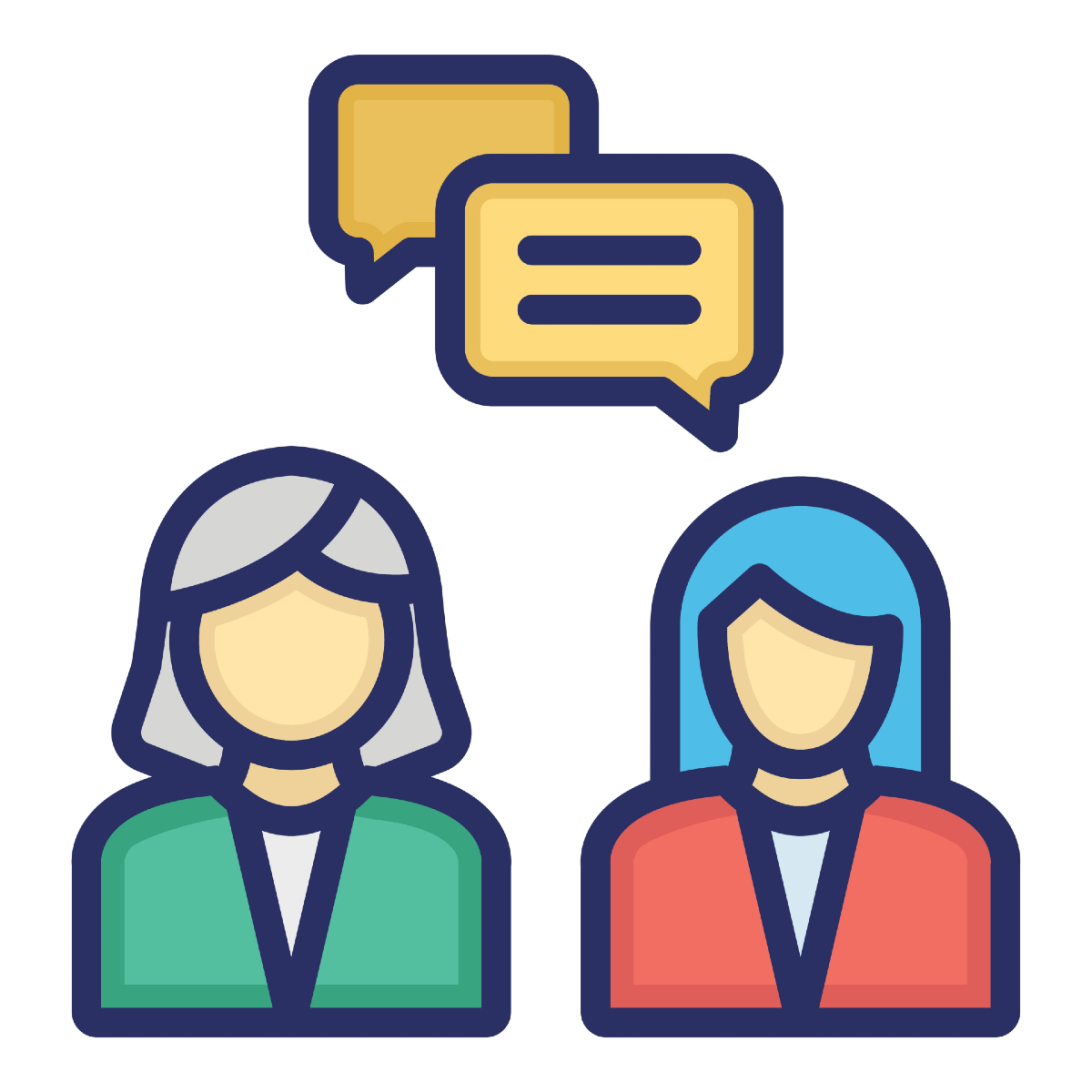	Brief information about how to structure professional communication about data gathered by mapping the patient health condition through the use of different communication tools and how to use these tools during clinical rotation
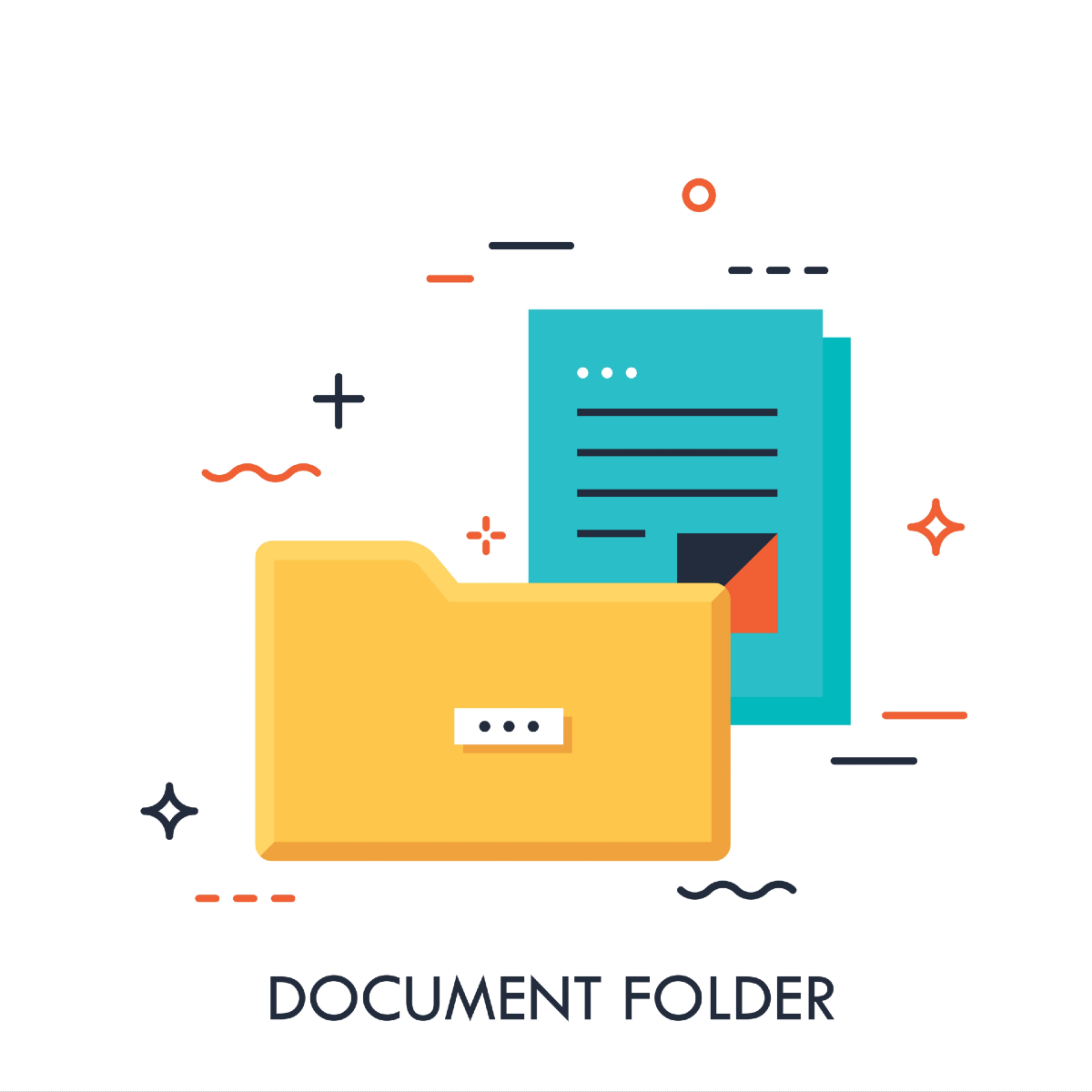	Information about how to structure the professional documentation of the information gathered through mapping the patient health condition by using B-PASs in the different documentation systems used in clinical practice
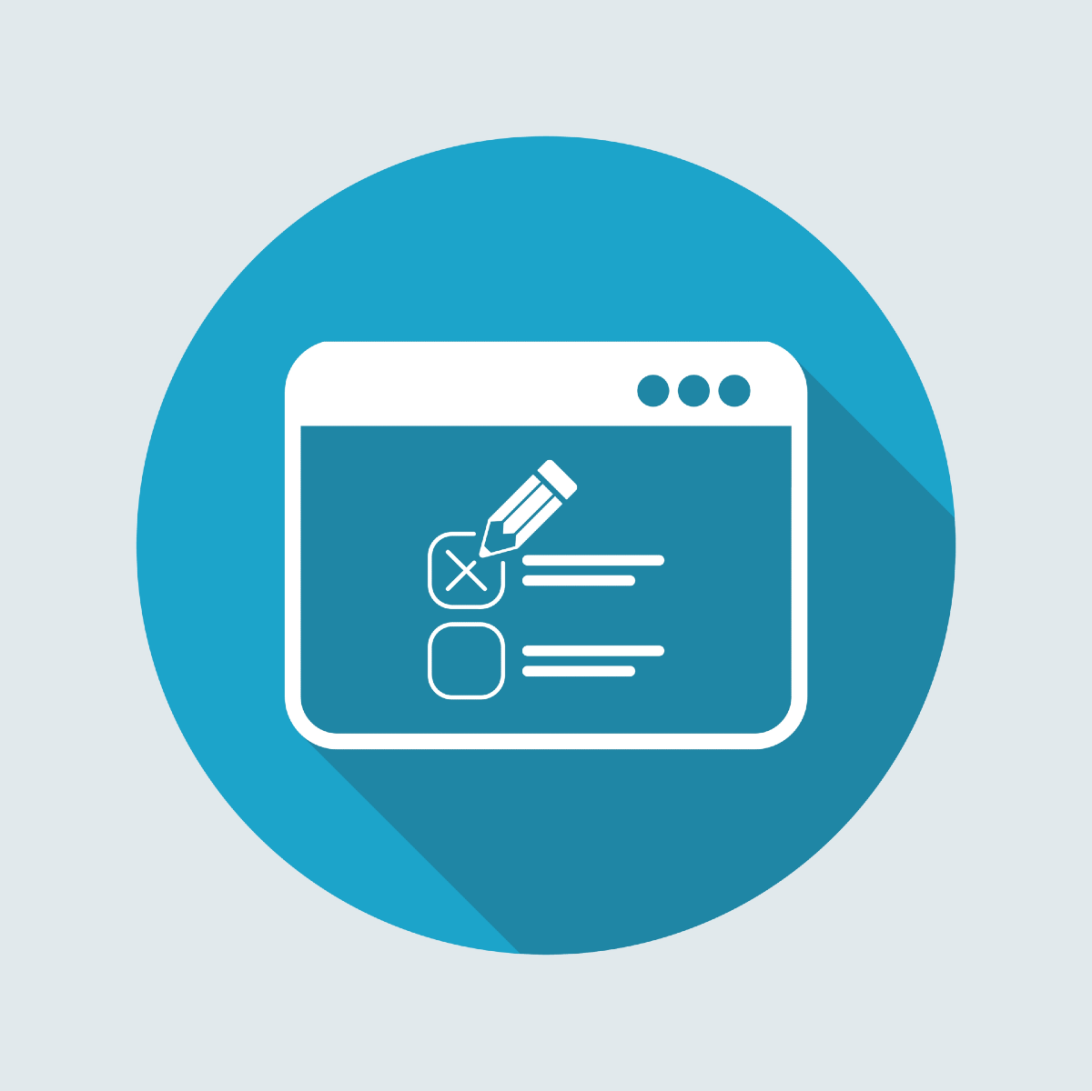	MCQ^d^ aiming to support students’ knowledge, to repeat and refresh bioscience knowledge, and to identify knowledge gaps
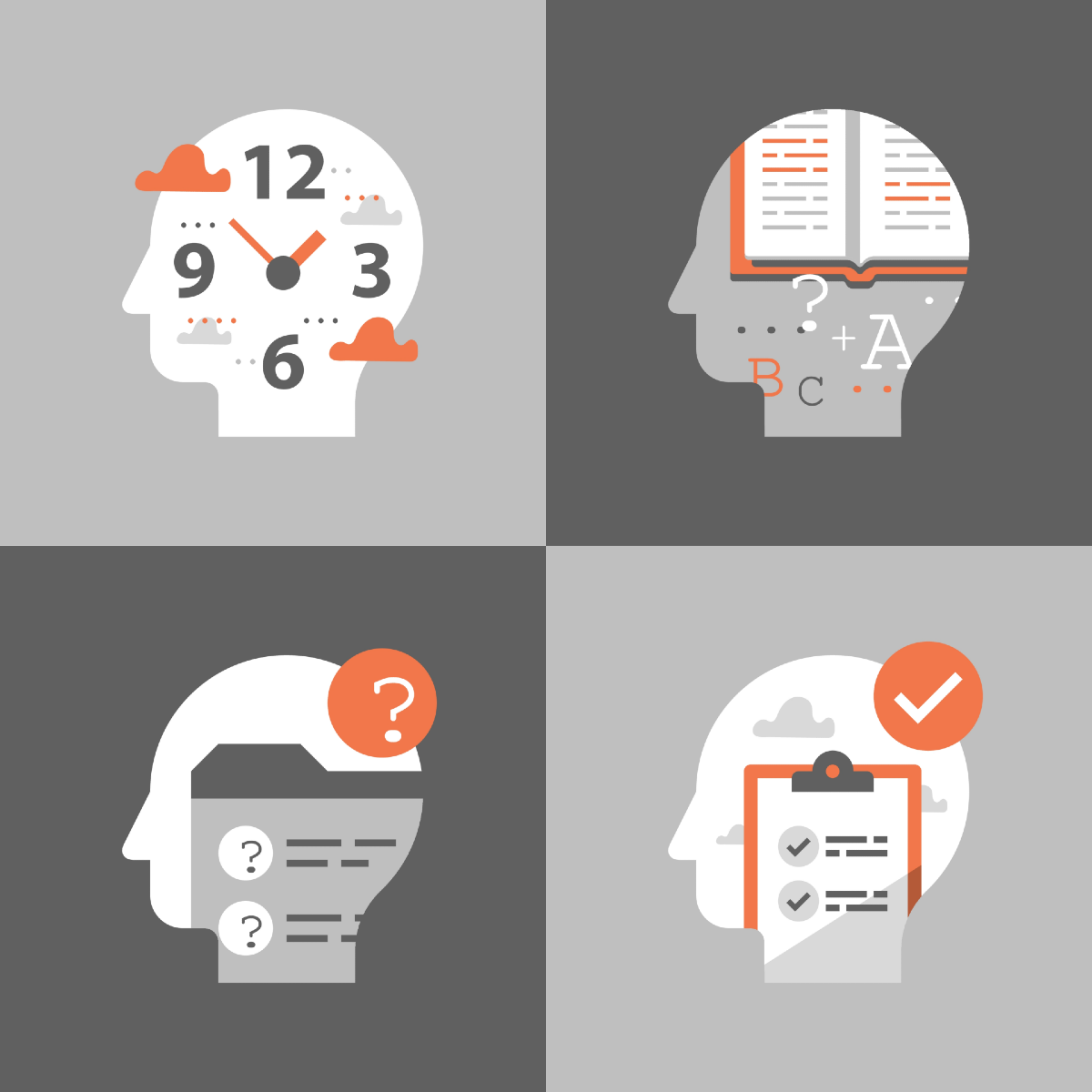	Description of a written assignment targeting reflection on the use of B-PASs. Feedback is given by fellow students (a peer review for the second- and third-year students) in which all parties are anonymous
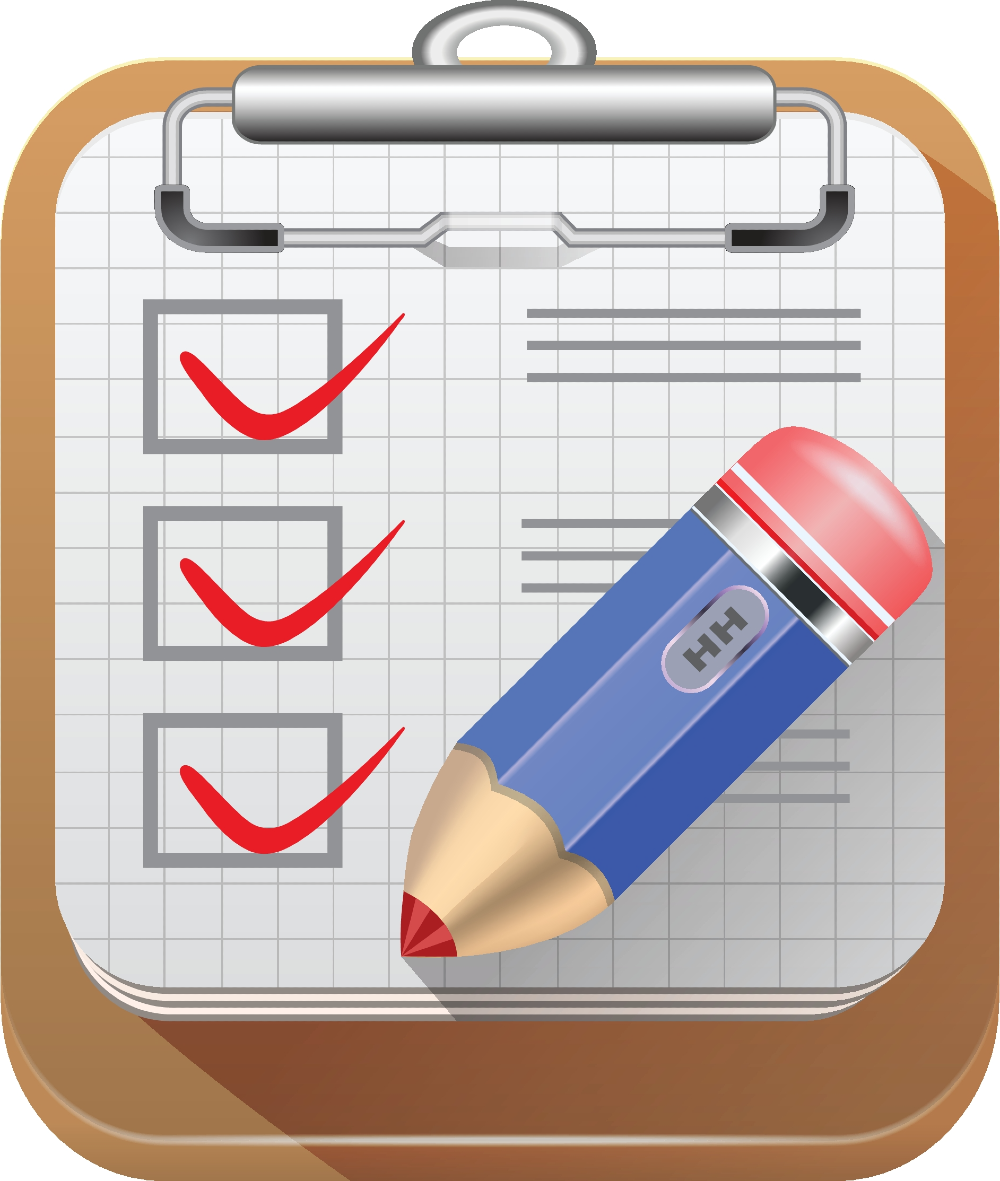	Checklists summarizing the elements of every focus in B-PASs (eg, respiratory system and neurological assessment). Can also be used when students use B-PASs in clinical rotation

^a^MOOC: massive open online course.

^b^B-PAS: basic physical assessment skill.

^c^RN: registered nurse.

^d^MCQ: multiple-choice question.

## Discussion

### Principal Findings

This study explored the use of the participatory design method to co-design a suite of mLearning tools specifically aimed at supporting the learning and performance of B-PASs and integration of human bioscience knowledge. As such, this study expands the use of mLearning with its explicit focus on the learning and application of practical skills, thus contributing to a better understanding of how different digital learning resources, individually and collectively, influence students’ learning and application of skills in nursing education.

### The Contribution of Digital Learning Resources to Enhance Learning of Skills and Knowledge

The most important findings of this study are regarding how the digital learning resources in the suite of mLearning tools enhanced learning processes related to specific categories of skills and knowledge. The digital learning resources stimulated the learning and application of B-PASs from a multidimensional perspective: from physical (motor) learning to the articulation of integrated theoretical knowledge and the enhancement of context-specific learning processes (Figure 5). Access to the suite of mLearning tools appears to have impacted the development of self-efficacy related to B-PASs in nursing students. As such, the students might perform B-PASs more frequently, and perhaps better, while also applying human bioscience knowledge in clinical reasoning processes. This is in line with other studies showing that students value access to different kinds of mobile resources to support and enhance learning [6,8,30,33-35]. The results of this study are also interesting, as although nursing students already use a variety of mobile apps—for example, drug calculators, medical dictionaries, handbooks, and clinical skills guides [6,8,33]—our suite of mLearning tools still seems to have added value. Nevertheless, there is a need for further exploration of how mLearning should be implemented across the nursing curriculum; this could be done by critically exploring which learning processes mLearning aims to support. Although mLearning alone might not be the best educational strategy in nursing education, in combination with other activities, it might represent a good educational approach [17,23].

Digital simulation with virtual patients was the preferred learning resource by students. The students not only valued the possibility of a new and innovative learning activity that the digital simulation offered but also highlighted that the involvement of faculty was crucial to facilitate good learning processes in this context. This facilitation role also involved creating a safe environment that allowed reflection *in* and *on* actions during the simulation session. The role of facilitation in digital simulation with a focus on B-PASs, both for the teacher and the preceptor, needs to be further developed. Although digital simulation as a part of the suite of mLearning tools is an individual learning activity, it also seems to be important to plan the digital simulation as a group activity to increase the learning outcome. As we have shown earlier and, in this study, because of a lack of adequate supervision with regard to B-PASs [17], the suite of mLearning tools plays an important role in supporting the learning and development of these skills and knowledge. Research shows that digital simulation alone can strengthen self-efficacy, performance of clinical skills, decision making [28], clinical reasoning processes [35,36], and nontechnical skills [37] of students. This indicates that including digital simulation with virtual patients contributes to better learning of skills, and that reflection contributes to the articulation of human bioscience knowledge, promoting clinical reasoning skills.

### Supporting Knowledge Transfer Between Different Learning Contexts for Skills Training

Another interesting finding of this study is how the co-design processes and the WS discussions revealed in what way digital learning resources can support knowledge transfer between different learning contexts. The suite of mLearning tools seemed to contribute to more seamless learning processes and thereby contributed to bridging the theory-practice gap in nursing, creating a more self-directed learning space for nursing students.

Lewin et al [37] argue that technology changes the boundaries of different types of learning (formal and informal) and learning spaces. This opens new ways to connect and combine different learning sites in higher education. As such, collaboration between universities and clinical settings may promote the creation of new knowledge and learning spaces, in which these institutions are equal partners in influencing learning and competence development in students. Chan et al [38] have termed this *seamless* learning: seamless learning processes enable students to navigate between different spaces and different roles, and to interact with different educational practices—for example, the university, the clinical setting, and the suite of mLearning tools [38]. As such, the suite of mLearning tools can promote transferability between learning contexts in nursing education. Therefore, further development and implementation should occur in close collaboration with students, the university, and the clinical setting.

However, the implementation of new and innovative teaching and learning strategies, such as the suite of mLearning tools, can face obstacles in both university and clinical settings. It is important for these obstacles to be identified and addressed to best support student learning. Attitudes of nursing staff, patients, and patients’ families toward the use of mobile devices may hinder their use of these devices [8,30,33,39]. The nursing students in our study reported similar experiences. It was also important for the students to be able to access the learning materials anywhere, at any time. Hsu and Hsiang [6] recommend that mLearning should support offline activity to secure a better end user experience. Taking this into consideration when designing and using a suite of mLearning tools in nursing education might enhance students’ experience of mobile technology as a pedagogical approach. This also highlights the importance of continuing to explore how mobile technology can be successfully implemented and support the teaching role of the faculty and the preceptors.

### Limitations

This study has several limitations that must be noted. The first-year students were absent in WS 3, which limited the opportunity to discuss with them the structure and the final content of the suite of mLearning tools. This needs to be explored in future studies. In addition, the students participating in this study might not necessarily represent the diversity of the university’s student population but rather those motivated students with the ability to participate in extracurricular activities. The use of the suite of mLearning tools should be tested by the entire student population to explore this further. Finally, the focus of the study was on the experience of the students with mLearning, not the perspectives of the faculty or preceptors, representing another limitation that should be addressed through future research.

### Comparison With Previous Work

The longitudinal design in this study offered opportunities to assess the different digital learning resources in 2 contexts: the university and clinical rotation. This process also strengthened the authenticity and the *here and now* experiences in the processes of assessing the feasibility and benefits of learning resources. O’Connor and Andrews [11] have shown the benefits of using a co-design approach with nursing students when designing an educational app supporting clinical skills in general and not specifically for B-PASs. Previous research in the field of mLearning related to PAS has only tested 1 app without involving students in the development process [6], whereas other studies have only focused on a single digital learning resource [24,26,28]. In contrast, this study explores multiple digital learning resources with high student involvement and identified the different impacts on students’ learning. The literature also highlights the need to reassess the educational strategies used to teach PASs to ensure that these skills are applied in a clinical setting [17,40,41].

### Conclusions

Students valued the invitation and opportunity to collaborate in co-design processes, thereby influencing the nursing education content. The longitudinal research design structuring the collaboration with students was essential to understanding what works and in which context. The nursing students viewed the suite of mLearning tools as beneficial for supporting B-PAS learning and their application during clinical rotation. Our findings indicate that one of the strengths of the suite of mLearning tools was its inclusion of all the different digital resources tested by the students; this variety in content met the different learning preferences and needs, enhancing B-PAS learning and human bioscience knowledge of the students. Therefore, the suite of mLearning tools may be a beneficial additional pedagogical strategy that supports knowledge and skills transfer between academic and clinical settings. Further studies are needed to explore different perspectives related to the use of mobile technology and mLearning (ie, faculty and preceptors), pedagogical strategies, and scaffolding learning material to better understand how mLearning can be used in nursing education.

## Abbreviations

B-PAS: basic physical assessment skill
LMS: learning management system
MCQ: multiple-choice question
mLearning: mobile learning
MOOC: massive open online course
PAS: physical assessment skill
WS: workshop
